# Editorial: Promoting Resilience Interventions for Mental Well-Being in Youth

**DOI:** 10.3389/fpsyt.2022.859546

**Published:** 2022-02-16

**Authors:** Silvia Gabrielli, Darko Roviš, Carmel Cefai

**Affiliations:** ^1^Digital Health Lab, Fondazione Bruno Kessler, Povo, Italy; ^2^Teaching Institute of Public Health of Primorsko Goranska County, Rijeka, Croatia; ^3^Centre for Resilience and Socio-Emotional Health, University of Malta, Msida, Malta

**Keywords:** resilience interventions, mental well-being promotion, youth interventions, prevention programs, resilience measurements

Children and adolescents face many challenges in today's fast changing society and constantly have to overcome increasing levels of adversity in order to achieve success. Unfortunately, difficulties in coping with emotional and social demands during development can have a negative impact on their ability to do so. This can result in impaired school achievements and lowered self-esteem, causing depression and anxiety whose consequences may persist into adulthood. Enhancing the ability of young people to cope with adversity by training in resilience skills has been the objective of several interventions and programs in the past years.

Resilience programs promote the development of protective and preventive factors, both at a personal and social level, that can help to overcome socio-emotional challenges in a positive and adaptive way. Past work has shown the importance of training resilience of youth by leveraging on relevant activities they typically perform in formal and informal learning environments. However, most research programs have focused on fostering social factors, such as family and school relationships, while fewer studies have analyzed the role of personal factors and digital health interventions in improving the resilience and coping skills of youth. More research is needed to understand the efficacy of evidence-based resilience programs in promoting mental well-being in youth, both in the short and long term.

Several contributions to this Research Topic have addressed the validation of resilience fostering programs for children living in socioeconomically disadvantaged contexts or belonging to vulnerable population. From the findings in Giordano, Caravita, et al. professionals are advised to design interventions by taking into considerations the multiple interaction between social-ecological resilience and avoidant coping strategies in the children adjustment. In the work of Kara et al. empirical findings from the implementation of the Bounce Forward program in the UK to improve children health, well-being and equity in the challenging town of Blackpool are presented, showing a positive effect of the program as a school-based intervention for prevention.

Fischmann et al. present an evaluation of long-term effects in two prevention programs for children-at-risk, growing up in deprived social environments, by focusing on child attachment representation as the primary outcome, as well as on self-reflective capacities of teachers taking care of these children. Fishbein et al., contribute an investigation to determine whether the positive effect of a socio-emotional learning program (PATHS) on children residing in a high poverty community were sustained over the course of two intervention years and an additional year when the intervention was no longer provided.

Weymeis et al. contribute a clinical trial aimed at validating the effectiveness of the TIME-IN intervention for improving emotional skills and emotional regulation in 8–12 years old children, by also reducing depressive symptoms.

Jones et al. provide inspiration for future interventions aimed at addressing the stressful condition of children with cancer, by presenting a literature review on three promising avenues for verbal therapy in pediatric oncology such as expressive writing, video narratives, and bibliotherapy exercises.

Lilja et al. investigate whether the Resilience Curriculum (RESCUR) program designed to foster the psychosocial development of children in early and primary education can scale-out of school to the social services sector, providing initial evidence that this can happen, by maintaining its implementation quality.

In another paper, Giordano, Cipolla, et al. present a transformative model of training service providers in resilience building, underlining the clients' strengths, and capacity for healing as well as contextually and culturally specific interventions.

Selameab and Mason contribute a study to identify potential structured institutional supports to graduate Public Health professionals from diverse communities to advance health equity, to adapt professional development supports in undergraduate Public Health programs and foster future workforce from communities impacted by health disparities.

Usher et al. carried out a scoping review of indigenous resilience in Australia, making use of a reflective decolonizing collective dialogue. The review reveals both the distinctive colonial characteristics of adversity experienced by Aboriginal people and the range of coping strategies and protective resources that support the development of resilience within different Aboriginal communities in Australia.

Li et al. undertook cross sectional study to examine the mediating role of resilience and self-esteem between life events and coping ways among rural left-behind adolescents in China.

A series of contributions to the Research Topic have addressed the investigation of resilience interventions for adolescents and undergraduate students. In de la Fuente, Santos, et al. the relations of resilience and positivity to coping strategies and engagement-burnout in undergraduate students is analyzed, in order to identify needs and propose therapeutic interventions for different student profiles. Another paper by de la Fuente, González-Torres, et al. present the findings of a cross-sectional study which investigated the role of resilience as buffering variable between the Big Five personality factors (according to their self-regulatory level) and academic stress amongst university students.

In Noh and Cho the psychological and physiological effects of the Mindful Lovingkindness Compassion Program (MLCP) intervention are investigated with university students in North Korea, showing that MLCP could be a promising intervention for alleviating self-criticism and increasing self-reassurance among self-critical individuals.

Grové contributes a brief research report on the co-design of a mental health and well-being chatbot to support young people in secondary schools or health care settings. This work can guide future design and deployment of such digital solutions to deliver resilience-based interventions for youth.

Long et al. present Be REAL (REsilient Attitudes and Living) program that has been shown to increase students' use of effective coping strategies, mindfulness, and sense of well-being.

Nartova-Bochaver et al. present a validation study on the 10-item Connor-Davidson Resilience Scale (CD-RISC-10) with a Russian youth sample, showing that this scale can be a valid, stable, and reliable instrument to use in future studies investigating the effectiveness of resilience interventions. Another validation study of Anderson et al. present a short version of Adolescent Resilience Questionnaire cutting the number of items to 49. Jonkman et al. examine the psychometric properties of the 28-item Child and Youth Resilience Measure (CYRM-28) among a sample of Dutch at risk youths to further assess the reliability of this instrument for its deployment in the Netherlands.

Finally, Afek et al. demonstrate the importance of inhibitory control for resilience and mental health in real life stressful situations, which could further inform the development of both resilience building and distress alleviating interventions among youth.

## Author Contributions

SG provided the general structure and contributed information about edited papers. DR revised the paper and contributed information about edited papers. CC revised the paper and contributed information about edited papers. All authors contributed to the article and approved the submitted version.

## Conflict of Interest

The authors declare that the research was conducted in the absence of any commercial or financial relationships that could be construed as a potential conflict of interest.

## Publisher's Note

All claims expressed in this article are solely those of the authors and do not necessarily represent those of their affiliated organizations, or those of the publisher, the editors and the reviewers. Any product that may be evaluated in this article, or claim that may be made by its manufacturer, is not guaranteed or endorsed by the publisher.

